# Transcriptomic and proteomic analysis of mouse radiation-induced acute myeloid leukaemia (AML)

**DOI:** 10.18632/oncotarget.9626

**Published:** 2016-05-26

**Authors:** Christophe Badie, Agnieszka Blachowicz, Zarko Barjaktarovic, Rosemary Finnon, Arlette Michaux, Hakan Sarioglu, Natalie Brown, Grainne Manning, M. Abderrafi Benotmane, Soile Tapio, Joanna Polanska, Simon D. Bouffler

**Affiliations:** ^1^ Radiation Effects Department, Centre for Radiation, Chemical and Environmental Hazards, Public Health England, Chilton, UK; ^2^ Faculty of Automatic Control, Electronics and Computer Science, Silesian University of Techology, Gliwice, Poland; ^3^ Helmholtz Zentrum München, German Research Center for Environmental Health GmbH, Radiation Proteomics Group, Institute of Radiation Biology, Neuherberg, Germany; ^4^ Radiobiology Unit, Institute for Environment, Health and Safety, Belgian Nuclear Research Centre (SCK•.CEN), Mol, Belgium; ^5^ Helmholtz Zentrum München, German Research Center for Environmental Health GmbH, Research Unit Protein Science, Neuherberg, Germany

**Keywords:** ionising radiation, acute myeloid leukaemia, mouse, gene expression, protein expression

## Abstract

A combined transcriptome and proteome analysis of mouse radiation-induced AMLs using two primary AMLs, cell lines from these primaries, another cell line and its *in vivo* passage is reported. Compared to haematopoietic progenitor and stem cells (HPSC), over 5000 transcriptome alterations were identified, 2600 present in all materials. 55 and 3 alterations were detected in the proteomes of the cell lines and primary/*in vivo* passage material respectively, with one common to all materials. In cell lines, approximately 50% of the transcriptome changes are related to adaptation to cell culture, and in the proteome this proportion was higher. An AML ‘signature’ of 17 genes/proteins commonly deregulated in primary AMLs and cell lines compared to HPSCs was identified and validated using human AML transcriptome data. This also distinguishes primary AMLs from cell lines and includes proteins such as Coronin 1, pontin/RUVBL1 and Myeloperoxidase commonly implicated in human AML. C-Myc was identified as having a key role in radiation leukaemogenesis. These data identify novel candidates relevant to mouse radiation AML pathogenesis, and confirm that pathways of leukaemogenesis in the mouse and human share substantial commonality.

## INTRODUCTION

Ionising radiation is a known leukaemogen, evidence from studies of the Japanese atomic bomb survivors indicate that acute myeloid leukaemia (AML) predominates [eg 1]. Mouse models of radiation-induced AML are available and there is substantial knowledge of the mechanisms that drive leukaemogenesis [2]. Furthermore, the relationship with human myeloid leukaemogenesis is becoming clearer, with PU.1 deregulation through mutation in the mouse or other regulatory pathways in human playing an important role [2]. The majority of mouse radiation-induced AMLs carry a deletion of one allele of the *Sfpi1* gene, the mouse homologue of human PU.1, and accompanying point mutations at codon 235 in the retained allele of the gene. Whether these two events are either necessary or sufficient for leukaemogenesis is not clear. There is at least one alternative pathway of leukaemogenesis in the mouse involving internal tandem duplication (ITD) mutations of *Flt3* [3], indicating that direct *Sfpi1* involvement is not necessary. Furthermore, some studies have suggested that point mutations are not rate limiting [4], possibly suggesting that *Sfpi1* deletion and point mutation are not jointly sufficient.

There is a need therefore to identify other common alterations associated with radiation-induced AMLs and to examine their relevance to AML pathogenesis. In this study we present a combined transcriptome and proteome analysis of a small set of mouse radiation-induced AMLs, comparing primary material, cell lines and an *in vivo* passaged cell line. Array based transcriptomics was combined with mass spectrometry-based proteomics and subsequent bioinformatics to provide a comprehensive and in depth analysis of alterations associated with leukaemogenesis and adaptation to *in vitro* culture.

The analysis strongly indicated that primary material is preferred for the identification of modifications related to disease pathogenesis. A set of seventeen genes/proteins is identified that is commonly altereded in these AMLs, several of these genes/proteins have roles in human leukaemogenesis and the gene set is able to distinguish human AMLs from normal control cells.

## RESULTS

**Figure 1:**
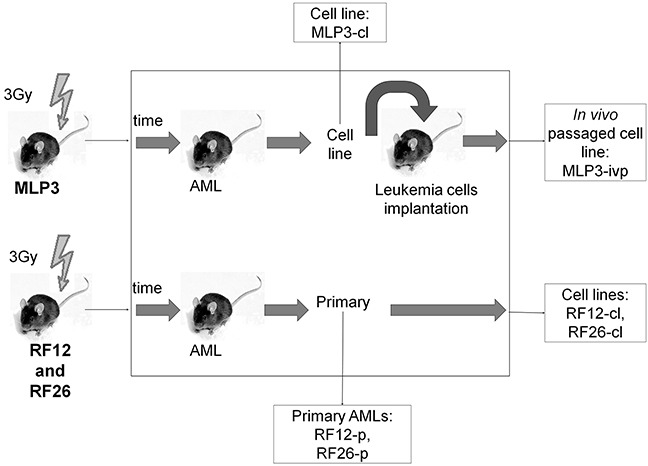
Schematic showing the origin of AML materials used in the study CBA/H mice were irradiated with 3 Gy x-rays, AMLs RF12, RF26 and MLP3 presented following several months latency. Spleen tissue from the mouse in which the AML arose was used as the source of primary AML material for RF12-p and RF26-p. These AMLs were also adapted to cell culture, to form RF12-cl and RF26-cl cell lines. Primary spleen tissue from MLP3 was not available but it was established as a cell line (MLP3-cl), the cell line was *in vivo* passaged to provide *in vivo* passaged MLP3 (MLP3-ivp).

**Figure 2:**
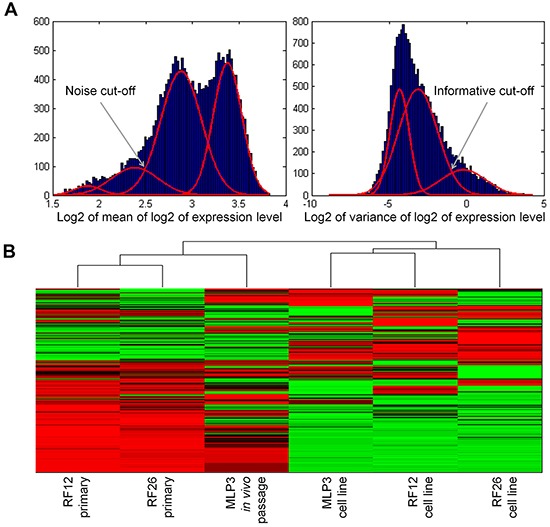
Unsupervised heat map of transcriptomics analysis, showing clustering of the primary and in vivo passaged samples and the AML cell lines **A.** normalisation procedure to eliminate transcripts below a specified noise threshold and above a specified variance threshold, led to construction of an unsupervised heatmap from 2152 transcripts **B**.

**Figure 3:**
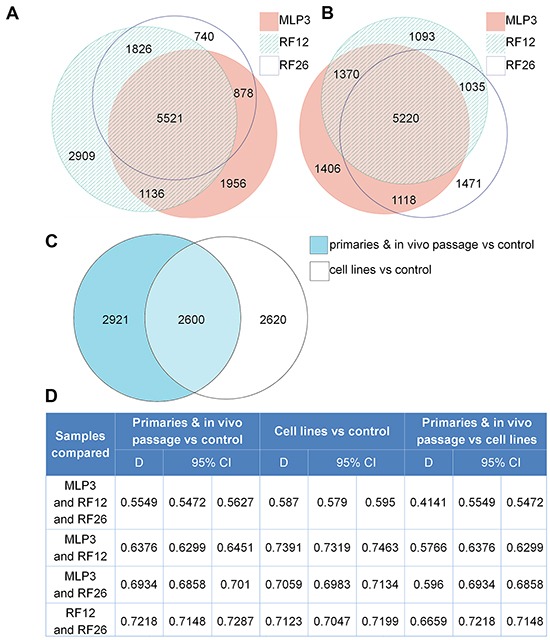
Transcriptome alterations in the primary and in vivo passaged samples, and cell lines **A.** Venn diagram summarising the numbers of transcripts altered in the two primary AMLs, RF12-p and RF26-p and MLP3-ivp by comparison with control HPSCs. **B.** Venn diagram summarising the numbers of transcripts altered in the three AML cell lines by comparison with the control HPSCs. **C.** Venn diagram summarising the numbers of transcripts commonly and uniquely altered in the three AML cell lines and two primary plus in vivo passaged samples. A subset of 2600 transcripts was found to be commonly altered in all materials compared to the control HPSCs. **D.** Dice index analysis of the degree of similarity between samples; see text for further discussion.

The experimental materials available (Figure 1) and results obtained allow for the identification of transcriptome and proteome changes associated with two primary leukaemias (RF12-p, RF26-p), three AML cell lines (RF12-cl, RF26-cl, MLP3-cl) and an *in vivo* passaged cell line (MLP3-ivp). The samples included in the current analysis were first characterised for *Sfpi1* status, given its important role in mouse radiation leukaemogenesis [2]. The primary AMLs and derived cell lines, RF12-p, RF26-p, RF12-cl and RF26-cl were found to be hemizygous for *Sfpi1* and carry point mutations in the retained allele such that the protein carried the R235C amino acid substitution. The MLP3 cell line is known to be deleted for both *Sfpi1* copies [5]. Figure 2 provides an unsupervised heat map of transcriptome modifications. This indicates that the two primary and one *in vivo* passaged AMLs show a high degree of similarity in the transcriptome modifications compared with the control sample, Lin-depleted bone marrow HPSCs. By contrast there are substantial differences in transcriptome between each cell line and its primary or *in vivo* passaged counterpart. The Venn diagrams (Figure 3A and 3B) summarise the findings; 5521 transcripts are commonly deregulated in the primary AMLs and *in vivo* passaged MLP3, 5220 transcripts are commonly deregulated in the three cell lines. While the number of transcripts deregulated in cell lines by comparison with primary and *in vivo* passaged material are similar, Figure 3C shows that only approximately half (2600) of the modifications are common in the two forms of material originating from the same primary AML, some 2620 modifications are specific to growth in *in vitro* culture. Supplementary Table S1 provides a full listing of the transcripts commonly altereded in AML cell lines, primary and *in vivo* passaged material.

Dice index analysis allows a quantitative overview of the degree of similarity between sets of samples – the closer to 1, the more similar groups of samples are. Figure 3D provides quantitative Dice index analysis for various comparisons of the deregulated transcripts. In 3-way comparisons between MLP3, RF12 and RF26, the primary/*in vivo* passaged materials are more different from cell lines (Dice = 0.4141) than either material is from control HPSCs, suggesting that caution is required in inferring changes in primary AML material from changes observed in cell lines. Pairwise comparisons tend to show a higher similarity between RF12-p and RF26-p than between MLP3-ivp and the RF12 & 26-p. In data not presented it has been observed that upregulated transcript changes tend to be more similar than down regulated transcripts.

To provide some insight into the functional significance of the transcriptional changes observed in AML materials, pathway analyses have been undertaken using the gene ontology and topGO database terms [6, 7]. This analysis identified significant representation of transcriptome changes in 28 GO terms in primary and *in vivo* passaged AMLs compared to control HPSCs. The comparison of cell lines and control indicated a much greater number of pathways being affected, represented by 110 GO terms, 24 of the 28 GO terms affected in primary (RF12-p, RF26-p) and *in vivo* passage (MLP3-ivp) material are also affected in the cell lines, these are listed in Table 1. The affected pathways included immune system processes, regulation of signalling, regulation of signal transduction, cytokine receptor activity and regulation of cell communication indicating substantial disruption of important regulatory processes in immune cells.

**Table 1:** List of the 24 pathways as defined by GO terms commonly affected in primary/in vivo passage and cell line AML materials compared to Lin-depleted bone marrow cells

#	GO_ID	Term name	ontology
**1**	**GO:0005623**	cell	CC
**2**	**GO:0044464**	cell part	CC
**3**	**GO:0008152**	metabolic process	BP
**4**	**GO:0010033**	response to organic substance	BP
**5**	**GO:0035556**	intracellular signal transduction	BP
**6**	**GO:0005488**	binding	MF
**7**	**GO:0043226**	organelle	CC
**8**	**GO:0003824**	catalytic activity	MF
**9**	**GO:0007275**	multicellular organismal development	BP
**10**	**GO:0002376**	immune system process	BP
**11**	**GO:0048583**	regulation of response to stimulus	BP
**12**	**GO:0009966**	regulation of signal transduction	BP
**13**	**GO:0044237**	cellular metabolic process	BP
**14**	**GO:0004896**	cytokine receptor activity	MF
**15**	**GO:0023051**	regulation of signaling	BP
**16**	**GO:0051179**	localization	BP
**17**	**GO:0070887**	cellular response to chemical stimulus	BP
**18**	**GO:0051234**	establishment of localization	BP
**19**	**GO:0032502**	developmental process	BP
**20**	**GO:0048519**	negative regulation of biological process	BP
**21**	**GO:0010035**	response to inorganic substance	BP
**22**	**GO:0010646**	regulation of cell communication	BP
**23**	**GO:0005737**	cytoplasm	CC
**24**	**GO:0044459**	plasma membrane part	CC

**Figure 4:**
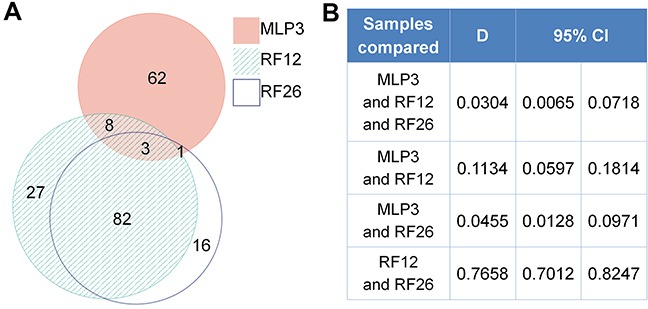
Proteomics analysis of primary and in vivo passaged samples **A.** Venn diagrams showing degree of similarity between the three samples all by comparison with control Lin-depleted bone marrow HPSCs, and **B.** quantitative Dice indices and 95% confidence intervals for pairwise comparisons. Note that the diagram was prepared using only proteins with complete data. In case of proteins with 1 or 2 measurements missing, the k-neighbour algorithm was used for data imputation and the protein was included into the analysis.

**Figure 5:**
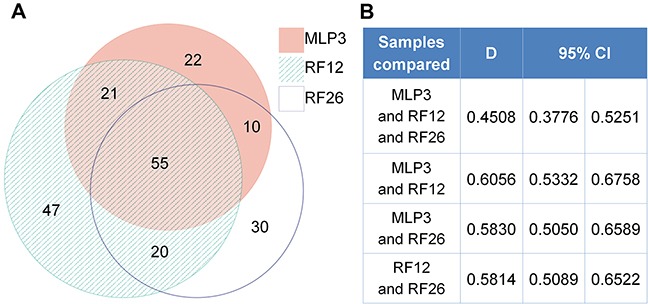
Proteomics analysis of AML cell lines **A.** Venn diagrams showing degree of similarity between the three samples all by comparison with control Lin-depleted bone marrow HPSCs, and **B.** quantitative Dice indices and 95% confidence intervals for pairwise comparisons.

The proteomics analysis is more limited in the number of distinct proteins that were detectable, the average number being around distinct 1,000 proteins. The number of identified and deregulated proteins in AML cell lines and primary cells were as follows: MLP3-cl (773 identified; 67 deregulated), RF12-cl (993; 111), RF26-cl (906; 102), MLP3-ivp (917; 151), RF12-p (1153; 180) and RF26-p (1153; 165). Nonetheless, commonly deregulated proteins were identified in primary and *in vivo* passaged materials (Figure 4A), RF12-p and RF26-p were notably similar at the level of deregulated protein as indicated by a Dice index of 0.77 (Figure 4B), however, only three proteins were commonly deregulated amongst the primary and *in vivo* passaged samples, Tln1, Tuba4a and Fkbp5. Substantially greater similarity of protein modification in cell lines compared to HPSC controls was apparent (Figure 5) with 55 commonly deregulated proteins identified (Table 2). Only the protein Fkbp5 was found to be commonly deregulated in all materials, ie the three cell lines, in vivo passaged MLP3 and the primaries.

**Table 2:** Proteins differentially expressed in the AML cell lines by comparison with Lin-depleted control bone marrow HPSCs

Entrez ID	Gene Symbol	Ensembl_Protein_ID	mean SLR Cell line vs Control		
			MLP3	RF12	RF26
**20832**	Ssr4	ENSMUSP00000002090	1.01	1.71	1.38
**67465**	Sf3a1	ENSMUSP00000002198	−3.63	−3.02	−4.15
**26395**	Map2k1	ENSMUSP00000005066	2.42	2.25	1.28
**21672**	Prdx2	ENSMUSP00000005292	−4.14	−3.35	−3.21
**11821**	Aprt	ENSMUSP00000006764	2.05	1.24	1.59
**16549**	Khsrp	ENSMUSP00000007814	−2.04	−1.27	−1.91
**13035**	Ctsg	ENSMUSP00000015583	2.35	3.54	1.12
**16993**	Lta4h	ENSMUSP00000016033	1.90	2.00	1.72
**15078**	H3f3a	ENSMUSP00000016703	−2.36	−3.79	−2.17
**17523**	Mpo	ENSMUSP00000020779	−6.47	−4.47	−1.85
**16416**	Itgb3	ENSMUSP00000021028	−4.00	−4.28	−5.29
**15212**	Hexb	ENSMUSP00000022169	2.02	2.23	4.34
**20655**	Sod1	ENSMUSP00000023707	−1.77	−2.62	−1.86
**16906**	Lmnb1	ENSMUSP00000025486	−1.25	−1.19	−1.59
**67988**	Tmx3	ENSMUSP00000025515	1.94	1.06	1.17
**14156**	Fen1	ENSMUSP00000025651	−1.58	−1.10	−1.55
**12359**	Cat	ENSMUSP00000028610	−1.80	−1.57	−2.61
**12349**	Car2	ENSMUSP00000029078	−8.43	−6.64	−6.64
**67103**	Ptgr1	ENSMUSP00000030069	3.06	2.87	3.97
**17025**	Alad	ENSMUSP00000030090	−3.98	−3.31	−4.09
**11669**	Aldh2	ENSMUSP00000031411	1.64	1.83	1.35
**11745**	Anxa3	ENSMUSP00000031447	1.32	1.85	1.70
**11674**	Aldoa	ENSMUSP00000032934	2.16	1.94	1.99
**12721**	Coro1a	ENSMUSP00000032949	−1.35	−2.95	−6.51
**12751**	Tpp1	ENSMUSP00000033184	−2.21	−2.50	−3.34
**11739**	Slc25a4	ENSMUSP00000034049	1.27	3.63	1.61
**12306**	Anxa2	ENSMUSP00000034756	4.09	2.39	2.13
**320011**	Uggt1	ENSMUSP00000037930	3.02	1.95	2.23
**233016**	Blvrb	ENSMUSP00000043092	−2.71	−3.88	−3.01
**18950**	Pnp	ENSMUSP00000043926	2.47	2.20	1.36
**18432**	Mybbp1a	ENSMUSP00000044827	2.56	3.00	4.45
**21825**	Thbs1	ENSMUSP00000044903	−1.73	−2.79	−3.45
**14870**	Gstp1	ENSMUSP00000047790	−1.79	−2.80	−1.32
**56307**	Metap2	ENSMUSP00000048285	−1.50	−1.40	−2.06
**14751**	Gpi1	ENSMUSP00000049355	2.35	1.73	2.12
**13861**	Epx	ENSMUSP00000050497	−3.50	−5.66	−5.48
**14104**	Fasn	ENSMUSP00000052872	3.31	3.26	2.09
**80838**	Hist1h1a	ENSMUSP00000062030	−2.22	−1.98	−2.73
**12332**	Capg	ENSMUSP00000063389	−1.43	−4.74	−1.64
**236539**	Phgdh	ENSMUSP00000064755	−1.47	−1.61	−1.03
**66222**	Serpinb1a	ENSMUSP00000075690	2.31	2.55	1.91
**13806**	Eno1	ENSMUSP00000079727	2.45	2.16	1.73
**18655**	Pgk1	ENSMUSP00000080302	2.54	2.07	2.12
**110208**	Pgd	ENSMUSP00000081141	2.34	3.99	1.49
**17105**	Lyz2	ENSMUSP00000089801	−2.47	−2.87	−3.65
**15288**	Hmbs	ENSMUSP00000095166	−3.50	−3.20	−4.87
**192176**	Flna	ENSMUSP00000098997	−1.60	−2.34	−3.12
**56431**	Dstn	ENSMUSP00000099461	2.96	2.98	2.13
**11637**	Ak2	ENSMUSP00000099664	1.66	1.03	1.17
**13382**	Dld	ENSMUSP00000106481	1.08	4.12	1.24
**12796**	Camp	ENSMUSP00000107653	−2.64	−5.08	−3.04
**108989**	Tpr	ENSMUSP00000112606	1.65	1.23	1.42
**14229**	Fkbp5	ENSMUSP00000116466	3.38	3.09	2.28
**15275**	Hk1	ENSMUSP00000118601	6.46	2.43	2.80
**11983**	Atpif1	ENSMUSP00000133099	−1.42	−2.34	−2.23

**Figure 6:**
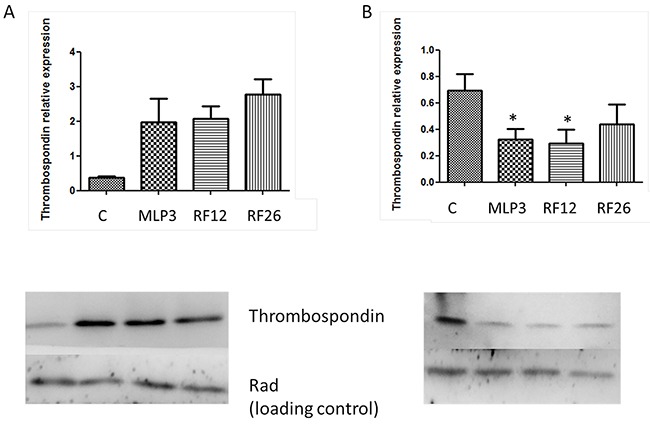
Western blot analysis of Thrombospondin 1 expression in the primary and in vivo passaged samples **A.** and the AML cell lines **B.** Lower panels show the blots including Rad50 (Rad), a loading control, with samples loaded in the following order from left to right: control (‘C’, normal mouse bone marrow HPSCs), MLP3, RF12, RF26. The upper panels provide histograms for quantitation of expression by image analysis, controls ‘C’ represent expression levels in normal mouse bone marrow HSPC, *indicate statistical significance at p<0.05 level compared to control.

The proteins altered in the cell lines included Thrombospondin 1 (Table 2). Western blot analysis was performed to confirm the mass spectrometry proteomics results (Figure 6). The results from cell lines are validated with Thps1 expression being around 1.5-2 fold lower compared to the control Lin-depleted bone marrow HPSCs. It can also be seen that in the primary AMLs and in vivo passaged MLP3 Thbs1 expression is approximately 2-fold higher than in the control. This suggests that growth conditions can influence the nature of expression changes observed.

**Figure 7:**
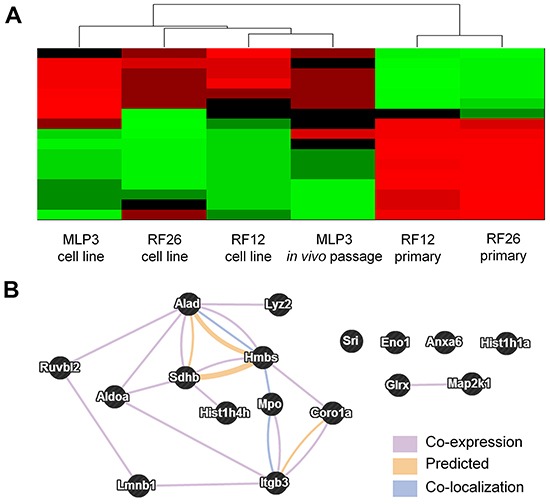
Multiomics analysis of combined transcriptome and proteome data **A.** heatmap showing relatedness between the samples, on the basis of these 17 transcripts/proteins it is possible to distinguish primary AMLs from cell lines. **B.** network analysis of the 17 identified proteins/genes (STRING db).

To integrate the transcriptome and proteome results a multiomic analysis was undertaken. This required the following steps (i) Identification of the transcripts representing the proteins included in proteomics analysis, (ii) For those transcripts, identification of those which differentiate between the cell lines (RF12-cl, RF26-cl, MLP3-cl) and the primary/*in vivo* passaged materials (RF12-p, RF26-p, MLP3-ivp) and the cell lines at p≤0.05 level (Bonferroni corrected), (iii) Amongst these significantly affected transcripts, identification of the represented proteins where the expression level is 2 fold up- or down- regulated – this results in 67 proteins/genes being identified, (iv) Of the 67, identification of those where transcript and protein are either both up- or down- regulated in the three cell lines and the primary and in vivo passaged materials or those where transcript and protein are up and down regulated respectively or vice-versa. The latter category was included as the analysis indicated that in primary AMLs there is a negative correlation between gene and protein expression, similar inverse relationships have been observed previously [eg 8, 9, 10]. This results in a set of 17 genes/proteins being identified that are deregulated at the gene and protein level in all AML materials examined. These genes/proteins allow RF12-p and RF26-p to be distinguished from RF12-cl, RF26-cl and MLP3-cl and *in vivo* passaged cell line, MLP3-ivp (Figure 7A, Table 3). Several of the genes/proteins are part of a network (Figure 7B) and the affected pathways as defined by KEGG and PANTHER terms are given in Table 4. Many of the transcripts/proteins have been implicated in human leukaemogenesis and the affected pathways include acute myeloid leukaemia and other cancer related pathways plus growth regulatory signalling, glycolysis and apoptotic signalling.

**Table 3:** Multiomics analysis of combined transcriptome and proteome data

Gene Symbol	Entrez GeneID	Gene Name
**Hist1h1a**	80838	histone cluster 1, H1a
**Anxa6**	11749	annexin A6
**Ruvbl2**	20174	RuvB-like protein 2
**Coro1a**	12721	coronin, actin binding protein 1A
**Sri**	109552	sorcin
**Mpo**	17523	myeloperoxidase
**Sdhb**	67680	succinate dehydrogenase complex, subunit B, iron sulfur (Ip)
**Lyz2**	17105	lysozyme 2
**Map2k1**	26395	mitogen-activated protein kinase kinase 1
**Lmnb1**	16906	lamin B1
**Itgb3**	16416	integrin beta 3
**Alad**	17025	aminolevulinate, delta-, dehydratase
**Glrx**	93692	glutaredoxin
**Hmbs**	15288	hydroxymethylbilane synthase
**Aldoa**	11674	aldolase A, fructose-bisphosphate
**Hist1h4h**	69386	histone cluster 1, H4h
**Eno1**	13806	enolase 1, alpha non-neuron

Identity of the 17 transcripts/proteins that are deregulated in all the AML materials analysed at both the gene and protein level.

**Table 4:** Pathways affected at the transcriptional and protein level in all analysed AML materials in the multiomics analysis

A) overrepresented KEGG pathways			
#	ID	KEGG pathway name	p-value
**1**	**mmu04066**	HIF-1 signalling pathway	0.0000845
**2**	**mmu04145**	Phagosome	0.0002630
**3**	**mmu00860**	Porphyrin and chlorophyll metabolism	0.0004380
**4**	**mmu00010**	Glycolysis / Gluconeogenesis	0.0011415
**5**	**mmu04919**	Thyroid hormone signalling pathway	0.0038027
**6**	**mmu04380**	Osteoclast differentiation	0.0041962
**7**	**mmu05034**	Alcoholism	0.0062702
**8**	**mmu05205**	Proteoglycans in cancer	0.0111167
**9**	**mmu04510**	Focal adhesion	0.0114341
**10**	**mmu04015**	Rap1 signalling pathway	0.0123001
**11**	**mmu04810**	Regulation of actin cytoskeleton	0.0124103
**12**	**mmu05206**	MicroRNAs in cancer	0.0171720
**13**	**mmu04320**	Dorso-ventral axis formation	0.0198055
**14**	**mmu05216**	Thyroid cancer	0.0229399
**15**	**mmu00030**	Pentose phosphate pathway	0.0237220
**16**	**mmu00020**	Citrate cycle (TCA cycle)	0.0245036
**17**	**mmu00051**	Fructose and mannose metabolism	0.0276238
**18**	**mmu05020**	Prion diseases	0.0276238
**19**	**mmu04151**	PI3K-Akt signalling pathway	0.0299092
**20**	**mmu05219**	Bladder cancer	0.0299578
**21**	**mmu05213**	Endometrial cancer	0.0407804
**22**	**mmu05223**	Non-small cell lung cancer	0.0438517
**23**	**mmu05221**	Acute myeloid leukemia	0.0446180
**24**	**mmu04370**	VEGF signaling pathway	0.0461490
**25**	**mmu04730**	Long-term depression	0.0469137
**26**	**mmu05210**	Colorectal cancer	0.0499664

B) overrepresented PANTHER pathways			
#	ID	PANTHER Pathways	P value
**1**	**P02746**	Heme biosynthesis	0.000054
**2**	**P02744**	Fructose galactose metabolism	0.008570
**3**	**P00024**	Glycolysis	0.000229
**4**	**P00020**	FAS signalling pathway	0.023400
**5**	**P00032**	Insulin/IGF pathway-mitogen activated protein kinase kinase/MAP kinase cascade	0.024100
**6**	**P05911**	Angiotensin II-stimulated signalling through G proteins and beta-arrestin	0.026900
**7**	**P00011**	Blood coagulation	0.040100
**8**	**P00056**	VEGF signalling pathway	0.040800
**9**	**P00054**	Toll receptor signalling pathway	0.040800
**10**	**P00010**	B cell activation	0.045600
**11**	**P00034**	Integrin signalling pathway	0.006930

**Figure 8:**
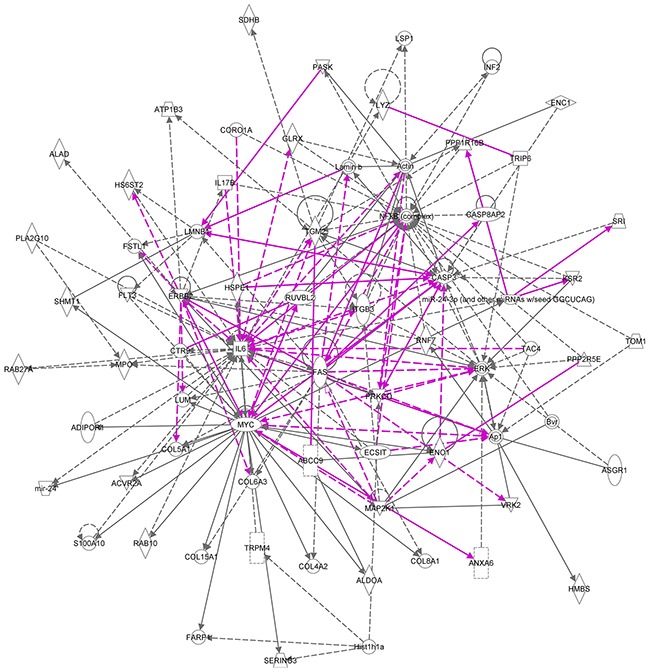
Merged ingenuity pathways for (i) cellular function and maintenance, cell death and survival, cellular compromise and (ii) connective tissue disorders, inflammatory disease, skeletal and muscular disorders All of the 17 member signature components are in this merged network with the exception of Hist1h4h. Solid lines imply direct relationships between proteins; dotted lines imply indirect interactions. Relationships are primarily due to co-expression, but can also include phosphorylation/dephosphorylation, proteolysis, activation/deactivation, transcription, binding, inhibition, biochemical modification. The purple lines represent the connections between the two most significant networks that were merged into one.

**Figure 9:**
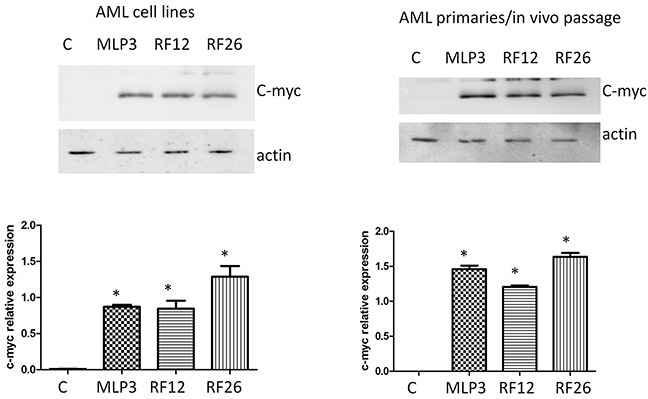
Western blot analysis of c-myc expression in the primary and in vivo passaged samples and the AML cell lines The upper panels show the blots including actin as loading control, with quantitation by image analysis in the histograms below. Lanes and bars marked ‘C’ represent the control normal mouse bone marrow HPSCs, *indicate statistical significance at p<0.05 level compared to control.

The top two networks identified using Ingenuity analysis, 1. Cellular Function and Maintenance, Cell Death and Survival, Cellular Compromise and 2. Connective Tissue Disorders, Inflammatory Disease, Skeletal and Muscular Disorders, in which most of the genes are present, can be combined (Figure 8). In these combined networks, MYC is major player, the overexpression of which was confirmed by Western blot analysis (Figure 9).

**Figure 10:**
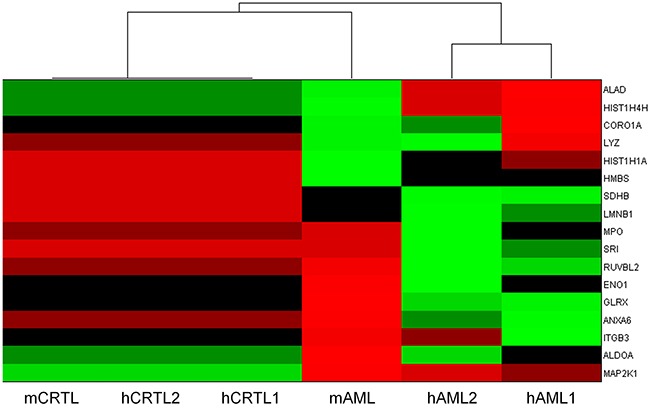
Heatmap summarising normalised transcriptome changes in expression of the 17 member signature genes in the mouse AML materials (mAML) examined in the current analysis and in human AMLs from publically available datasets (hAML1 – karyotypically normal AMLs, hAML2 – monosomy 7 AMLs) plus respective controls (mCTRL, hCTRL1, hCTRL2)

To explore the wider use of the 17 gene/protein signature and its ability to distinguish other AML samples, an analysis of the expression of the genes in two human AML datasets was carried out. The human data sets were generated from analysis of bone marrow from four karyotypically normal AMLs compared to granulocyte macrophage progenitors (GMPs) from six healthy normal control donors and bone marrow from AMLs with monosomy of chromosome 7 compared to healthy control donor GMPs respectively. An overall summary of this analysis is shown as a heatmap (Figure 10). The 17 gene signature correctly clusters the human and mouse control samples and is able to discriminate each AML sample set from the control. Furthermore each AML group is distinct from each other. Mean centred box plots relating to each individual gene and sample are provided in Supplementary Figure S1.

## DISCUSSION

This transcriptome and proteome analysis included within the study design materials from primary AMLs (RF12-p, RF26-p), derived cell lines (RF12-cl, RF26-cl), an independently derived and long-established AML cell line (MLP3-cl) and *in vivo* passaged MLP3 (MLP3-ivp). The datasets allowed identification of transcript and protein alterations associated with leukaemogenesis and those associated with adaptation to growth *in vitro* and the impact of an *in vivo* growth environment. The transcriptome analysis was more comprehensive (21266 transcripts) than the proteomic analysis (1005 proteins). The results obtained strongly indicate that primary AML material is best used to identify common changes of relevance to leukaemogenesis, adaptation of primary material to growth *in vivo* has substantial impacts on the transcriptome and proteome. Broadly speaking if cell lines are selected for analysis, only approximately 50% of detected changes may be of relevance or leukaemogenesis (see Figure 3). Further evidence that adaptation to cell culture has a significant effect on the transcriptome comes from the observation that primary and *in vivo* passaged materials are more similar to the control HPSC population (obtained directly from bone marrow without *in vitro* culture) than are the *in vitro* cultured cell lines (Figure 2). Further Dice index analysis of transcriptome data (Figure 3) and Western blotting (Figure 5) has shown that expression changes in cell lines can differ from those in primary material. The inclusion of the MLP3 cell line and MLP3-ivp was particularly informative. The Western analysis (Figure 6) indicates that the expression Thsp1 differs under the two growth conditions, indicating an important role for the microenvironment in determining gene and protein expression. The unsupervised transcriptome heatmap (Figure 1) clearly groups MLP3-ivp more closely to the primary AMLs RF12-p and RF26-p than its in vitro passage counterpart, MLP3-cl, adding further weight to the argument that growth microenvironment is an important determinant of gene expression levels, and that alterations are not necessarily cell intrinsic.

The transcriptome analysis identified 24 pathways that are commonly deregulated in all AML materials. The affected pathways (Table 1) include immune system processes, regulation of signalling, regulation of signal transduction, cytokine receptor activity and regulation of cell communication indicating substantial disruption of important regulatory processes in the AML cells.

Partly because of the more limited range of proteins analysed, the commonly deregulated proteins were fewer in number with just 3 being commonly deregulated in the three primary and *in vivo* passaged samples, Fkbp5, Tln1 and Tuba4a and just one, Fkbp5, commonly deregulated in all AML materials (cell lines, *in vivo* passaged MLP3 and primary AMLs). Fkbp5, FK506 binding protein 5, is an immunophillin that is most highly expressed in T-lymphocytes and the thymus. The FK506 binding proteins are involved in multiple cellular processes including protein folding, receptor signalling, protein trafficking, transcription, apoptosis and T-cell activation [11]. Over 200 regulatory microRNAs are predicted to interact with Fkbp5 (Mouse Genome Database, accessed September 2015). Tln1, Talin 1 bridges between vinculin and integrins providing a link between the cytoskeleton and extra-cellular matrix, the reduced expression of Tln1 likely relates to the release of AML cells from the bone marrow into the peripheral circulation that occurs characteristically late in the pathogenesis of radiation AML in the mouse. Tuba4a is a tubulin, also indicating modification of the cytoskeleton. Amongst the wider group of 55 proteins that were commonly deregulated in the three AML cell lines (Table 2) are several involved in proliferative signalling, apoptosis, myeloid cells and leukaemia. Ten of these proteins are also identified as part of the 17 gene/protein signature, characteristic of all AML materials identified through the multiomic analysis, and some of these are considered further below.

Bringing the data together in a multiomic analysis has confirmed that there is significant similarity in the pathways deregulated at the transcriptome and proteome levels. Perhaps most importantly, this analysis indicated a small set of 17 genes/proteins (Table 3) that is characteristically deregulated in all the AML materials examined in this study and therefore represent a potential gene/protein signature of mouse AML. The expression levels of these genes/proteins can further distinguish the two primaries (RF12-p, RF26-p) from the remaining cell line – derived samples and indicate greater similarity amongst *in vitro* cultured samples than between *in vitro* and *in vivo* cultured samples. This point emphasises the need to examine primary AML materials to be more confident to identify key changes related to the disease pathogenesis. The affected pathways (Table 4) include many related to cancer, and specifically acute myeloid leukaemia, along with some relating to growth regulation, apoptotic signalling and the haemopietic system. Interestingly many of the genes/proteins included in the 17 member ‘signature’ have been found to have roles in human and mouse leukaemogenesis. In particular Coronin 1 (Coro1a), a member of the evolutionarily conserved coronin protein family involved in a variety of cellular processes, is highly expressed in all leukocytes. In mice and human, genetic inactivation of Coro1a results in immuno-deficiencies and it was recently shown that human PU.1 is a direct transcriptional regulator of CORO1A in acute promyelocytic leukaemia and acute myeloid leukaemia [12]. We also identified pontin/RUVBL1 a gene which is upregulated by AML1-ETO and generated by one of the most frequent chromosomal rearrangements in human AML (translocation t(8;21)(q22;q22)) and reported to participate in the oncogenic growth of t(8;21) cells [13]. The percentage of MPO positive leukemic cells is a simple and highly significant prognostic factor in AML patients [14]. MPO as well as lamin B1 (LMNB1) genes carry polymorphisms which predict risk of relapse of childhood acute lymphoblastic [15]. Integrin Beta 3 (Itgb3) is essential for leukaemogenesis but dispensable for normal haematopoiesis hence suggesting that Itgb3 signalling pathway is a potential therapeutic target in AML [16]. Succinylacetone (SA; 4,6-dioxoheptanoic acid), a specific inhibitor of delta-aminolevulinic acid dehydrase (ALAD), leading to growth inhibition of leukaemia cells, [17] is associated with an important pathway in mouse erythroleukaemia cells [18]. In mouse, erythroleukaemias are associated with Friend virus infection where a clonal leukaemia develops through the proviral insertional activation of Spi1/Pu.1 leading to overexpression of PU.1 [19, 20]. Porphyria is a disease which can be caused by mutations in ALAD as well as the HMBS gene hydroxymethylbilane synthase (Hmbs). Interestingly Porphyria is often diagnosed in erythropoietic protoporphyria in association with haematological malignancy [21]. Two other genes identified are members of histone cluster 1 and a PcG methylation of the histone cluster 1 (including Hist1h1a and Hist1h4h) has been identified as an epigenetic marker of AML [22].

The mitogen-activated protein kinase kinase 1 (MAP2K1) is a member of the dual specificity protein kinase family which acts as a mitogen-activated protein kinase (also known as extracellular signal-regulated kinase ERK). Mutations in FLT3 kinase which are frequent in AML patients and were identified in radiation-induced mouse AML [3], affect the extracellular signal-regulated kinase ERK1/2 [23]. Sorcin (Sri) is highly expressed in the heart and in the brain, and overexpressed in many cancer cells in general and in AML [24]. The subunit B Succinate dehydrogenase (SDHb) expression is modified in AML [25]. Levels of serum Lysozyme (Lyz2) are sometimes used as an aid for diagnostic AML subtyping and prognosis in AML [26]. Glutaredoxin (GRX) is a redox-regulating protein putatively associated with neoplastic process in a human leukaemia HL-60 cell line [27]. There are some reports on aldolase (Aldoa) activity in B chronic lymphocytic leukaemia and hyperaldolasaemia has been detected in patients with AML [28]; although literature screening failed to find a known direct involvement of Enolase 1 (Eno1) in AML, it is a tumour marker without recognised role in cancer [29]. Finally, the last gene identified, Annexin A6 (Anxa6) belongs to a family of calcium- and phospholipid-binding proteins in which increased expression leads to the constitutive activation of extracellular signal-regulated kinase ERK. Interestingly Annexin A1 was reported to be a PU.1 target in leukaemic cells [30].

Our analysis also highlights the potential importance of the MYC oncogene (Figures 8, 9); MYC is known to be commonly overexpressed in AML due to trisomy 8/15 (human/mouse), FLT3-ITD mutation, or gene amplifications (double minute chromosomes) [31]. C-Myc also rapidly induces acute myeloid leukaemia in mice when expressed in the bone marrow [32]. In radiation-induced mouse AML, significantly higher expression of c-myc was previously reported especially in the PU.1-deficient (deletion of one copy and R235 point mutation) AMLs [33] and interestingly in therapy-related AML, abnormalities of chromosomes 5 and/or 7 accounted for 76% of all cases with an abnormal karyotype and it has been reported that AML with a −5/del(5q) have a higher expression of c-MYC [34]. Furthermore, c-myc has been identified as an AML driver mutation in a retrovial transduction/transplantation screen in mice [35]. These data confirm that AMLs are characterised by deregulation of transcriptional networks that control the lineage specificity of gene expression. Collectively, the data suggest that this signature has potential value as a biomarker relevant for AML early detection and diagnosis, treatment or prediction of response to therapy. The potential wider application of the signature and its use on human as well as mouse material has been validated using two human AML datasets (Figure 10). Furthermore, while most of the 17 transcripts show similar levels of expression in the human AML datasets, four (CORO1A, ITGB3, RUVBL2, SRI) are expressed differentially between the two human AML datasets (Supplementary Figure S1). These may be useful for AML sub-typing; an issue that would benefit further investigation.

In summary, a combined transcriptome and proteome analysis has been carried out on two primary AMLs, the cell lines from these primaries, one additional AML cell line and an *in vivo* passage of that cell line. The data were analysed individually and in a combined multiomics analysis. This has revealed a small set of genes/proteins, many related to cancer development and leukaemia specifically, commonly affected in all materials. These provide an insight into potential novel candidates relevant to pathogenesis of radiation-induced AML in the mouse, and confirm that pathways of leukaemogensis in the mouse and human share substantial commonality in the pathways affected. The proteomics analysis suggested that Talin 1 might be involved in the late stage release of leukaemic cells from the bone marrow into the peripheral circulation and subsequent tissue infiltration. A general finding is that in AML cell lines, approximately 50% of the transcriptome changes are related to adaptation to cell culture, and in the proteome this proportion is higher. It is also clear that the growth microenvironment plays a major role in determining gene and protein expression patterns in these AML samples. Therefore it is more efficient to use primary materials to search for additional target genes and proteins related to radiation AML pathogenesis.

## MATERIALS AND METHODS

An overview of the materials used is provided in Figure 1.

### rAML induction experiments

The protocol for AML induction generated from CBA\H mice whole body irradiated has been described previously [36]. Briefly, mice were whole-body 3 Gy irradiated at 12–15 weeks of age with 250 kVp X-rays at a dose-rate of 0.887 Gy/min (MRC, Harwell, Oxon,UK). AMLs were diagnosed using the criteria described in the Bethesda Proposals for Classification of Non-Lymphoid Neoplasms in mice [37]. Mice were examined daily for signs of illness and euthanised with a rising concentration of CO_2_. Animals found to have increased white blood cell counts in the peripheral blood film and displaying splenomegaly or hepatosplenomegaly upon dissection were treated as potential AMLs. Samples of spleen were either stored at −70°C in RNAlater (Ambion,Austin, US) for nucleic acid extraction or disaggregated and used for flow cytometry or cytogenetics. All cases defined as AML had a rapid onset,with ≥20% immature forms/blasts found when spleen cell samples were analysed by flow cytometry, and a white blood cell count above that of controls (controls: approx.5–10 × 10^6^/mL). Flow cytometry analysis furthermore established that cases of AML are further defined by cells surface marker expression [38]. Animals were bred and handled according to UK Home Office Animals (Scientific Procedures) Act 1986 and with guidance from the local ethical review committee on animal experiments.

### RF12 and RF26 cell lines

Cell lines were established from disaggregated AML spleen cells. Cells (3-5 × 10^6^) were washed with PBS (1200 rpm, 5 mins) and resuspend in 10 ml culture medium (RPMI 1640 with 10 % fetal bovine serum, FBS, and 10ng/ml IL3, Sigma). Cell suspensions were transferred to T25 vented flasks and placed in humidified incubator at 37°C and 5% CO_2_. Cells were maintained in these conditions for approximately 6 weeks, and split in to new flasks with fresh medium when confluent. IL3 was then removed from the medium, if the culture continued to grow independently of IL3, it became defined as an established cell line. It has been found that only a minority of AMLs adapt to growth in cell culture under this protocol.

### MLP3 cell line

Derived from a primary AML induced in a CBA/H mouse from Drs Emmy Meijne and Rene Huiskamp (NRG, Petten, Netherlands). Cell lines were maintained in RPMI 1640 plus 20% foetal bovine serum, with added L-glutamine, penicillin and streptomycin at standard concentrations (Invitrogen, UK). Cultures were maintained in a humidified incubator at 37°C and 5% CO_2_.

### AML passage

AML cells from spleen cells or cell lines grown in culture were passaged in to CBA recipient mice to obtain large amounts of material for genetic and protein analysis.

Cell suspensions were prepared by washing homogenised spleen or cells from *in vitro* culture twice in Iscove's Modified Dulbecco's Medium (IMDM medium, Sigma-Aldrich Co, St Louis, MO, USA). 1 × 10^6^ cells in 250 ml IMDM medium were injected intraperitoneally in to each of 4 recipients. Recipient mice were monitored at least daily for presentation of AML. Any mice displaying symptoms of AML (decreased movement, hunched posture, rapid breathing, pallor, enlarged spleen on palpation), or any other significant deviation from normal were euthanized by a rising concentration of CO_2_. Tissues and cells were obtained and stored as for primary AMLs.

### Sfpi1/PU.1 copy number assessment

The protocol used for chromosome 2 deletion identification has been described previously [38, 39].

### Mutation status of Sfpi1/PU.1

DNA was extracted from AML using a DNeasy Blood and Tissue kit (Qiagen) according to manufacturer's instructions, exon 5 of Sfpi1/PU.1 was amplified by PCR and sequenced as described by Suraweera et al [40].

### Immunomagnetic cell separation

To obtain HPSC populations (Lin–or Lin–Sca-1+c-Kit+(LSK) cells), bone marrow cells were flushed from femora and tibias of 8 donor mice either exposed to 3 Gy X-rays 7–9 days beforehand or from unirradiated controls. Lin-depleted cells were selected using the Mouse Hematopoietic Progenitor Enrichment Kit (Stem Cell Technologies, Grenoble, France) according to the manufacturer's instructions.

### RNA extraction and transcriptome analysis

Total RNA was extracted from flash frozen cells using the AllPrep DNA/RNA/protein Mini kit (Qiagen, Hilden, Germany), quality-controlled using a 2100 BioAnalyzer (Agilent, Santa Clara, CA, USA) and quantified using a Nanodrop 2000c spectrophotometer (Thermo Scientific, Wilmington, USA). Only samples with an RNA Integrity Number ‘RIN’ >8.0 were used for further gene expression analysis. Using the WT Expression Kit (Ambion Inc, Austin, TX, USA), cDNA was prepared from 10 μg of purified cRNA, originally synthesised and purified from 0.25 μg of total RNA following the manufacturer's instructions. The cDNA (2.75 μg) was then used for fragmentation and labeling using GeneChip Terminal Labelling Kit (Affymetrix, Santa Clara, CA, USA). Using GeneChip Hybridization, Wash and Stain (Hybridization module) (Affymetrix, Santa Clara, CA, USA), and Hybridization controls (Affymetrix, Santa Clara, CA, USA), fragmented and labelled cDNA was hybridized to Mouse Gene 2.0 ST Arrays (Affymetrix, Santa Clara, CA, USA). After hybridization under orbital rotation for 16 h at 45°C, arrays were washed and stained using GeneChip Hybridization, Wash and Stain Kit (Stain module) (Affymetrix, Santa Clara, CA, USA) according to the manufacturer's instructions. Finally, arrays were scanned immediately using Affymetrix GeneChip Scanner GS 3000.

### Proteomic analysis

#### ICPL Labelling and one-dimensional gel electrophoresis (1-DE)

Proteins from AML cell lines and AML primary cells were extracted using 1% Triton-X100 (40 mM Tris, pH=7.6) buffer. The extracted proteins were precipitated with the 2D clean-up kit (GE Healthcare) following the manufacturer's instructions. The pellets were resuspended in ICPL lysis buffer (SERVA) and triplicate aliquots of 100 μg of protein were labelled with ICPL reagents (SERVA) as described previously [41, 42]. After labelling, equal amounts of light and heavy labelled samples were combined and separated by 12% SDS gel electrophoresis [43] before staining with colloidal Coomassie [44]. The gel lanes were cut in 3 slices and subjected to in-gel digestion. Prior to digestion, proteins were destained with 50 mM NH_4_HCO_3_ in 30% acetonitrile (ACN). In-gel digestion was performed overnight with trypsin of sequencing grade (SERVA Electrophoresis GmbH, Germany) using a total protein to enzyme ratio of 50:1 in 10 mM NH_4_HCO_3_. Peptides were extracted and acidified with 1% formic acid for subsequent mass spectrometry analysis.

#### LC/MS/MS analysis

The digested peptides were separated by reversed phase chromatography (PepMap, 15 cm × 75 μm ID, 3 μm/100Å pore size, LC Packings) operated on a nano-HPLC (Ultimate 3000, Dionex) with a nonlinear 170 min gradient using 2% acetonitrile in 0.1% formic acid in water (A) and 0.1% formic acid in 98% acetonitrile (B) and eluted with a flow rate of 250 nl/min. The gradient settings were: 0-140 min: 2-30% B, 140-150 min: 31-99% B, 151-160 min: stay at 99% B and equilibrate for 10 min at starting conditions. The nano-LC was connected to a linear quadrupole ion trap-Orbitrap (LTQ Orbitrap XL) mass spectrometer (Thermo Fisher, Bremen, Germany) equipped with a nano-ESI source. The mass spectrometer was operated in the data-dependent mode to automatically switch between Orbitrap-MS and LTQ-MS/MS acquisition. Survey full scan MS spectra (from m/z 300 to 1500) were acquired in the Orbitrap with resolution R = 60,000 at m/z 400 (after accumulation to a target of 1,000,000 charges in the LTQ). The method used allowed sequential isolation of up to ten most intense ions depending on signal intensity, for fragmentation on the linear ion trap using collision-induced dissociation at a target value of 100,000 ions with a normalised collision energy of 35 % and an activation time of 30 ms. Minimum signal intensity required was 200, isolation with 2 amu and default charge state 2. Precursor masses were selected in a data-dependent manner. High resolution MS scans in the Orbitrap and MS/MS scans in the linear ion trap were performed in parallel.

Target peptides already selected for MS/MS were dynamically excluded for 30 seconds. General mass spectrometry conditions were: electrospray voltage, 1.25-1.4 kV; no sheath and auxiliary gas flow. An activation Q-value of 0.25 and activation time of 30 ms were also applied for MS/MS. The acquired MS/MS spectra were searched against the Ensembl *Mus musculus* database using an in-house version of Mascot (release 62 with 54,576 sequences). A version of MASCOT (Matrix Science, version 2.3.02 with a number of residues of 26 203 053) was used with the following parameters: MS/MS spectra were searched with a precursor mass tolerance of 10 ppm and a fragment tolerance of 0.8 Da. MASCOT scores are probability-based MOWSE score: −10xLog(P), where P is the probability that the observed match is a random event. Scores >34 indicate identity or extensive homology; p <0.05. One missed cleavage was allowed. Carbamidomethylation was set as fixed modification. Oxidised methionine and the heavy and light ICPL labels of lysines as well as heavy and light ICPL labels of the protein N-terminus were set as variable modifications.

Data processing for protein identification and quantification of ICPL pairs was performed using Proteome Discoverer version 1.3 (Thermo Fisher) as described before [45]. Proteome Discoverer (Thermo Scientific) software performs automated statistical analysis of the results and uses unique peptides to calculate accurate relative protein quantification. All proteins showing significance p < 0.05 and fold-change >2 or <0.5 in Proteome Discoverer and Perseus software tool [45] were considered as deregulated.

#### Immunoblotting

For the validation of protein expression changes by immunoblotting, 20 μg protein extract was separated on 8% or 12% SDS polyacrylamide gels according to Laemmli [43]. Proteins were transferred to nitrocellulose membranes (GE Healthcare) using a semidry blotting system at 100 mA for 90 min. Membranes were saturated for one hour with 5% advance blocking reagent (GE Healthcare) in TBS (50 mM Tris.HCl, pH 7.6 and 150 mM NaCl) containing 0.1% Tween 20 (TBS/T). Blots were incubated overnight at +4°C with antibodies against either cMYC (Santa Cruz Biotechnology, Inc.) or thrombospondin (Abcam).

After washing three times in Tris-buffered saline/Tween 20 TBS/T, blots were incubated for one hour at room temperature with horseradish peroxidase-conjugated anti-mouse or anti-goat secondary antibody (Santa Cruz Biotechnology) in blocking buffer (TBS/T with 5% w/v advance blocking reagent). Immunodetection was performed either with ECL advance Western blotting detection kit (GE Healthcare) following standard procedures. The protein bands were quantified using ImageQuant 5.2 software (GE Healthcare) by integration of all pixel values in the band area after background correction and normalised to the loading control, RAD50 (GeneTex, Taiwan) or actin (Santa Cruz Biotechnology, Inc., US).

#### Bioinformatics, statistics and data handling

RMA algorithm [47] with Dai annotation [48, version 19, November 2014] was used for normalisation of transcriptomic data. Gaussian Mixture Model based algorithm [49] was applied for both noise level detection and filtration of uninformative features. Two-way and one-way ANOVA followed by Tukey-Kramer tests for pairwise comparisons were performed to verify the hypothesis on mean value equality independently on cell type and AML passage. The analysis was carried out separately for hypotheses on up- and down- regulation. Benjamini-Hochberg correction for multiple testing was applied to avoid high level of false discoveries [50]. Generalised Dice index [51] and its 95% confidence interval were used as a measure of set similarity. Functional analysis of differentially expressed genes was performed using Bioconductor *TopGO* package (Alexa A. and Rahnenfuhrer J.: topGO: Enrichment analysis for Gene Ontology. R package version 2.22.0) with *parentchild* method and hypergeometric test for overrepresentation [52].

In case of proteomic data, only proteins detected in at least four of six experimental conditions were considered. The missing data were imputed by k-nearest neighbours algorithm combined with linear regression technique [53]. The proteins with significant fold change greater than 2 or lower than 0.5 were recognised as deregulated.

Integration of multiomic data was performed by mapping of Protein Ensembl identifers to Gene Entrez identifiers with the use of Bioconductor *biomaRt* package [54]. Spearman correlation coefficient was calculated to measure association between gene and protein expression levels. Hierarchical clustering UPGMA (Unweighted Group Method with Arithmetic Mean) bottom-up algorithm with standardised Euclidean metric and average linkage was applied to create the samples' dendrogram [55]. Validation of the 17 gene/protein signature utilised the publically available human AML datasets, GSE35008 (four AMLs with normal karyotype), and GSE35010 (six AMLs with monosomy of chromosome 7) [56, 57] - before performing UPGMA hierarchical clustering of averaged mouse and human samples, due to the different platforms on which gene expression was measured, expression levels for each of the 17 transcripts were transformed into z-scores within each dataset using the respective healthy control as a reference.

## SUPPLEMENTARY FIGURE AND TABLE




