# Supplementary material for: Transcriptomic and proteomic analysis of mouse radiation-induced acute myeloid leukaemia (AML)
Source: Oncotarget. 2016 May 26;7(26):40461–80. doi: 10.18632/oncotarget.9626 (PMC5130020; doi:10.18632/oncotarget.9626)
Supplement: Supplementary file 1 [file oncotarget-07-40461-s001.pdf]

Transcriptomic and proteomic analysis of mouse radiation–induced acute myeloid leukaemia (AML)

SUPPLEMENTARY FIGURE AND TABLE

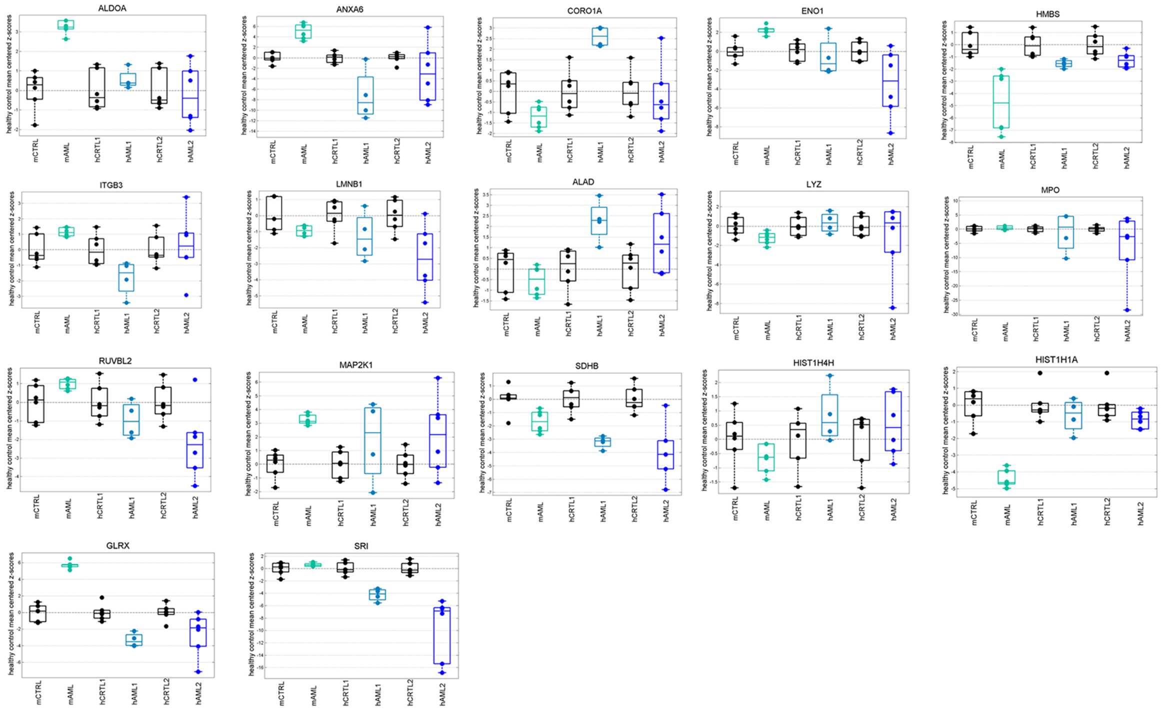

Supplementary Figure S1: Box plots showing normalised, mean centred z-scores of the expression of each of the 17 member gene signature in the mouse AML samples studied in the current analysis, 4 AMLs with normal karyotype (hAML1) and the respective control (hCTRL1) and 6 AMLs monosomic for chromosome 7 (hAML2) and their controls (hCTRL2).

Supplementary Table S1: Table of the 2600 genes commonly deregulated in cell lines and primary/in vivo passaged samples. (Excel file).

See Supplementary File 1
